# Impact of Dietary Supplementation of Probiotics on Cecal Microbial Ecology, Immune Response, and Meat Quality of Muscovy Ducks

**DOI:** 10.3390/microorganisms14010182

**Published:** 2026-01-14

**Authors:** Ahmed Mohammed, Eman Negm, Nadim Amarin, Sherief Sayed, Ahmed Soliman, Hussam Askar, Shaymaa Yusuf, Asmaa Adel Rayan

**Affiliations:** 1 Department of Behavior and Management of Animals, Poultry and Aquatics, Faculty of Veterinary Medicine, Assiut University, Assiut 71526, Egypt; 2 Department of Physiology, Faculty of Veterinary Medicine, Assiut University, Assiut 71526, Egypt; 3 Poultry Business Unit Director EMEA and APAC, United Animal Health Inc., Sheridan, IN 46069, USA; nadim.amarin@unitedanh.com; 4 Department of Food Hygiene Safety and Technology “Meat Hygiene”, Faculty of Veterinary Medicine, Assiut University, Assiut 71526, Egypt; 5 New Life Inc., Cairo 4451801, Egypt; 6 Department of Zoology, Faculty of Science, Azhar University, Assiut 71524, Egypt; 7 Department of Immunology and Microbiology, Faculty of Veterinary Medicine, Assiut University, Assiut 71526, Egyptasmaa-adel@aun.edu.eg (A.A.R.)

**Keywords:** Muscovy ducklings, probiotic, welfare, immunity, meat quality

## Abstract

Probiotics represent a beneficial approach to boost the welfare, health, and meat quality of poultry. One hundred and twenty one-day-old male Muscovy ducklings were divided among 24 floor pens (five ducklings per pen). The pens were randomly distributed among one of four dietary treatments with six replicates (G-C) without any supplementation of probiotics; (G-A) was supplemented with 0.4 g/kg of Amnil^®^; (G-M) was supplemented with 0.5 g/kg of M-Mobilize^®^; and (G-A-M) was supplemented with 0.4 g/kg of Amnil^®^ (1–30 day) and 0.5 g/kg of M-Mobilize^®^ (31–60 day), respectively. The results indicated that BW at day 60 was improved in (G-A) birds compared with (G-C) ones, IL-6 was decreased in (G-A) and (G-A-M) in liver and spleen in comparison with (G-C) (*p* < 0.05), but no differences were observed between (G-C) and (G-M) (*p* > 0.05); IL-10 was decreased in all the probiotic-fed ducklings compared with (G-C) birds in the spleen (*p* < 0.05), and IL-10 was decreased in the (G-A) birds compared with the other treatments in the liver (*p* < 0.05). Probiotic-fed birds showed a higher enumeration of *Lactobacillus* spp. compared to (G-C) group (*p* < 0.05). In addition, the (G-M) group showed improved breast meat flavor, general acceptability, and water-holding capacity (WHC%) compared to (G-C) group (*p* < 0.05). These results suggest that the probiotic supplement (G-A), could be a good management tool for improving Muscovy ducks’ health and production and further research is needed to improve meat quality traits.

## 1. Introduction

Poultry farming is a vital component of modern agriculture, playing a crucial role in the production of eggs and meat, and contributing significantly to the overall food industry through high-quality meat, lipids, vitamins, minerals, and proteins [1]. The return of poultry investment for fast growth and increased weight gain can be improved through several strategies, such as intensification, hybridization, and selection [2]. Undesirable consequences such as stress, disease, metabolic disorders, fat deposition, leg problems, and downgraded meat quality can be increased while selecting other traits. Nutrition plays a significant role in modifying the performance, immunity, well-being, and meat quality of poultry without undesirable consequences [2].

The gut microbiota has garnered much interest over the years due to its significant effects on poultry health maintenance. A broad diversity of microorganisms shapes this gut microbiota, which maintains health through the enhancement of the immune system, improvement in nutrient absorption, or prevention of pathogen colonization [2]. The preservation and restoration of this microbiota is a key factor in maintaining a healthy intestinal microbiome and related physiological homeostasis [2].

Antibiotics have been utilized as growth promoters in poultry for many decades [1]. However, drug residues in meat products have contributed to the rapid emergence and spread of antimicrobial resistance (AMR) in bacterial populations [3]. Antimicrobial resistance would be responsible for the deaths of an additional 10 million people/annum by 2050 [4]. Financially, antimicrobial resistance’s estimated impact on global gross domestic product (GDP) is a loss of $100–210 trillion [5]. The search for alternative strategies in poultry production has been crucial. Probiotics are found as promising alternatives [2]. Probiotics are nonpathogenic organisms (yeast or bacteria, especially lactic acid bacteria) in poultry diets that can exert a positive influence on the host’s health [1]. The theory is that live microorganisms in a diet in the form of a supplement improve the microbial balance of the intestinal tract [2].

The inclusion of probiotics in poultry diets improves microbiota balance in the gastrointestinal track, inhibits the growth of pathogenic bacteria, promotes food digestion and nutrient resorption, boosts immune function, and improves welfare indicators [2,6,7].

Several probiotics, each consisting of different bacterial combinations, have been investigated in broiler chickens and ducks, with varied findings [8,9]. These conflicting results may be partially due to variations in the probiotic compositions (i.e., bacterial strains) [10]. Despite the widespread use of probiotics in humans and other animals, the impact of probiotics on Muscovy ducklings’ meat quality and its relation to cecal microbial ecology and immune response remain largely unexplored [2,7,8,9]. In poultry, most of the probiotic investigations have been focused on broiler chickens and ducks’ production [1,8]. However, studies on the use of probiotics in duck’s health and welfare are still scarce. Therefore, the objective of this study was to investigate the effects of two compositions of dietary supplements of probiotics (Amnil, a combination of *Bacillus subtilis* and *Bacillus licheniformis* strains, and M-mobilize, a combination of yeast extract, *Bacillus subtilis*, *Lactobacillus plantarum*, *Pediococcus*, and *Acidilactici strains*) and a possible synergistic effect by feeding Amnil (from 1 to 30 day) and M-mobilize (from 31 to 60 day) on the modification of cecal microbial ecology and related immunity, meat quality, and welfare in Muscovy ducks. We hypothesized that the probiotic dietary supplements would improve the health status of Muscovy ducks via regulation of gut microbiota and modification of immune response and meat quality.

## 2. Materials and Methods

All procedures and bird handling were approved by the Animal Care and Use Committee of the Faculty of Veterinary Medicine, Assiut University, Egypt (Number: 06/2025/0383).

### 2.1. Probiotics

The commercial probiotics (Amnil^®^ and M-Mobilize^®^, United Animal Health, Sheridan, IN, USA) utilized in this experiment contained microbial strains, i.e., Amnil^®^ contained *Bacillus subtilis* and *Bacillus licheniformis* strains, and M-Mobilize^®^ contained yeast extract, *Bacillus subtilis*, *Lactobacillus plantarum*, and *Pediococcus acidilactici* strains.

### 2.2. Animals and Housing

One hundred and twenty male Muscovy ducklings were obtained from a commercial hatchery (El-Salam Inc., Cairo, Egypt) and transferred to a temperature-controlled room in the Animal and Poultry Behavior and Management Research Unit, Faculty of Veterinary Medicine, Assiut University, Egypt. The birds were weighed and assigned to 24 floor pens (110 cm × 110 cm per pen) (five ducklings per pen) with a similar average initial body weight. The ducklings were maintained at a temperature of approximately 32–34 °C in the first week by using a digital thermometer at the level of the bird’s height, and decreased by 3–5 °C per week until it reached 19–20 °C at four weeks as the ducklings were fully feathered until the end of the experiment [8].

### 2.3. Dietary Treatments

The 24 pens were randomly assigned to four dietary treatments with six replicates of five ducklings: a control (G-C) without any supplementation of probiotics; (G-A) was supplemented with 0.4 g/kg of Amnil^®^; (G-M) was supplemented with 0.5 g/kg of M-Mobilize^®^; and (G-A-M) was supplemented with 0.4 g/kg of Amnil^®^ (1–30 day) and 0.5 g/kg of M-Mobilize^®^ (31–60 day), respectively. The probiotic dietary treatments were supplied from day 1 to 60 and made by the step-up procedure. In brief, the respective amount of probiotic product was mixed with a small amount of the regular diet as a small batch, and then incorporated with a larger amount of the diet gradually, until the total amount of each of the particular diets was homogeneously mixed [2] (Table 1).

### 2.4. Tonic Immobility Test

On day 59, two ducklings were selected and brought to an adjacent room. One hand was put on the duckling’s chest and the other over its head; the duck was restrained for 5 s and placed on a cradle upside down in a wooden table. Then, the researcher removed his hands and walked a few steps away from the bird, and avoided eye contact. After complete immobility of the birds, the test was performed for 5 min, and the test was terminated after the bird was righteous and duration of immobilization was recorded. The examiner repeated the restraining of the bird if it righted itself for less than 5 s five times [9].

### 2.5. Sample Collection

On day 60, two ducks per pen were randomly selected. Sodium pentobarbital was used to anesthetize the ducks by injection in the brachial vein (30 mg/kg of body weight). The cervical dislocation method was used to euthanize the birds, and then the liver, spleen, and cecal contents (approximately 1 g) were collected. All tissue samples were kept at –80 °C until they were analyzed. After collection of the tissue samples, the ducks were eviscerated manually, and the carcasses were washed and allowed to drain for 10 min. After draining, the right breast of each duckling was separated and stored at 3 ± 0.5 °C for 24 h, following the traditional farm fresh meat procedure [2].

### 2.6. Immune Response

Liver and spleen concentrations of interleukin-6 (IL-6), interleukin-10 (IL-10), and tumor necrosis factor alpha (TNF-α) were detected using the commercial duck enzyme-linked immunosorbent assay kits according to the company’s instruction (Sinogeneclon Co., Ltd., Hangzhou, China).

### 2.7. Microbial Analysis

In brief, 1 g of each cecal content sample was diluted in 9 mL of buffered peptone water (Neogen Corporation, Lansing, MI, USA), and then 10-fold serial dilutions up to 10^−7^ were performed. Eosin methylene blue agar (Fisher Scientific/Becton, Dickinson Co., Sparks, MD, USA) and Rogosa agar (Fisher Scientific/Becton, Dickinson Co.) were used for enumeration of *Escherichia coli* and total lactobacilli by inoculation of 10 μL from each of the serial dilutions for 24 h at 37 °C aerobically. Then, the colonies were counted and the results were expressed as units per gram of the sample [10].

### 2.8. Meat Quality Analysis

#### 2.8.1. Meat pH

The pH values of the cooled breast muscle samples were measured using a calibrated pH meter (SD 50 pH meter, Lovibond^®^, Dortmund, Germany), with a two-point calibration at pH 7 and pH 4. A 10 g portion of each sample was homogenized in 90 mL of distilled water at 25 °C using a stomacher (Seward 400, TodMed, OH, USA). The pH sensor was directly immersed into the homogenate, and the pH value of the breast meat was recorded [11].

#### 2.8.2. Sensory Analysis

Briefly, post-rigor breast muscle samples were boiled until fully cooked (approximately 10–15 min), then cut into portions and kept warm at 65 °C in Petri dishes until evaluation (within 10 min). A sensory panel, consisting of four members from the Food Hygiene Department, Faculty of Veterinary Medicine, Assiut University, assessed the samples. Panelists evaluated color, taste, juiciness, chewiness, flavor, and overall acceptability using a nine-point hedonic scale. To minimize sampling order bias, the order of sample presentation was randomized [11].

#### 2.8.3. Meat Water-Holding Capacity %

Water-holding capacity (WHC%) was assessed based on the percentage of meat weight lost under applied pressure. Approximately 0.3 g of meat cubes, taken from the same region of each sampled breast muscle, were placed between two filter papers and two ceramic plates. A 5 kg weight was applied to the top plate for 3 min. The difference in sample weight before and after compression was recorded to determine the released water percentage (RW%). WHC was then calculated using the following formula: WHC (%) = 100 − RW% [11].

#### 2.8.4. Meat Cooking Loss

The cooking loss (CL) of breast meat samples was determined following the method described by [11], with slight modifications. Briefly, raw breast muscle samples were first weighed, then boiled until fully cooked (approximately 15 min), allowed to cool to room temperature, and weighed again. Cooking loss was calculated as the percentage difference between the initial (raw) and final (cooked) weights of the samples.

### 2.9. Statistical Analysis

A complete randomized design was used for the present study. Data were subjected to one-way analysis of variance (ANOVA, SAS Institute, Cary, NC, USA) and the pen was considered as the experimental unit (*n* = 6). The fixed effect was the probiotic treatments, and the subsample was the two birds of each pen. The Shapiro–Wilk test was used for analyzing the normality of the data. When a significant difference was found, means were further compared using the Tukey–Kramer test. Statistical significance was set at *p* < 0.05.

## 3. Results

### 3.1. Body Weight

**Figure 1:**
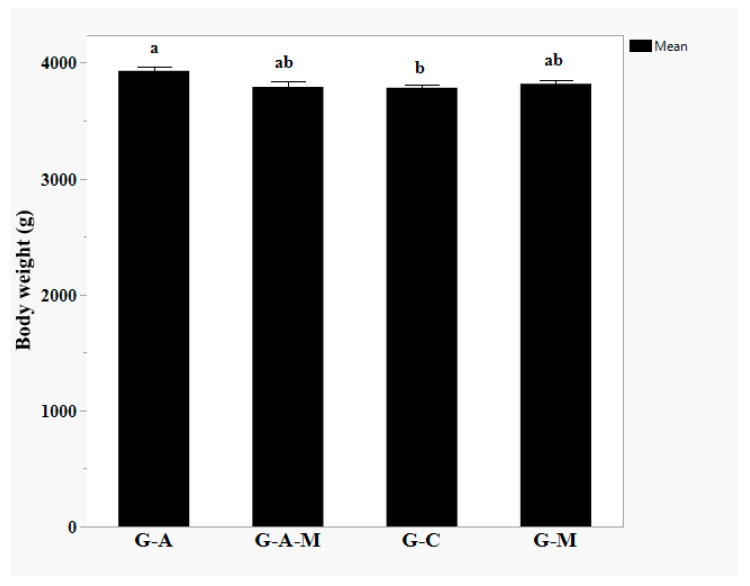
Effect of dietary supplementation of multi-strain probiotic supplements on the BW of Muscovy ducks. ^a, b^ Least square means with different superscripts differ significantly (*p* < 0.05). Dietary treatments containing a control (G-C) without any supplementation of probiotics, G-A; supplemented with 0.4 g/kg of Amnil^®^, G-M; supplemented with 0.5 g/kg of M-Mobilize^®^, and G-A-M; supplemented with 0.4 g/kg of Amnil^®^ (1–30 day) and 0.5 g/kg of M-Mobilize^®^ (31–60 day), respectively, *n* = 12 per treatment (pen was considered the experimental unit, all two birds/pen were used to measure meat quality parameters and averaged for analysis).

Compared to controls, (G-A)-fed birds had a heavier BW, but no differences were observed in the (G-M) and (G-A-M); (*p* = 0.0385, Figure 1).

### 3.2. Tonic Immobility Test

The probiotic supplementation effects on the tonic immobility test are presented in Table 2. The tonic immobility test was not affected by the probiotic supplementation (Normal average is 200 to 250 s [12] (*p* > 0.05)).

### 3.3. Immune Response

The probiotic supplementation effects on the immune response in the liver and spleen are presented in Table 3. IL-6 was decreased in (G-A) and (G-A-M) in liver and spleen in comparison with (G-C), but no differences were observed between (G-C) and (G-M), and IL-10 decreased in the probiotic-fed ducks compared with (G-C) birds in the spleen; IL-10 was decreased in the (G-A) birds only compared with the other treatments in the liver (*p* < 0.05). There was no statistical effect of the probiotic supplementation on the concentration of TNF-α in the liver and spleen (*p* > 0.05).

### 3.4. Cecal Microbiota

The probiotic supplementation effects on the cecal microbiota are presented in Table 4. Compared to the control, the probiotic-fed birds had a higher count of *Lactobacilli* spp. (*p* < 0.05), but there was no statistical effect of the probiotic supplementation on the levels of coliforms (*p* > 0.05).

### 3.5. Meat Quality

The probiotic supplementation effects on meat quality are presented in Table 5. Compared to controls, breast meat from (G-M) ducks had better outcomes in the general sensory analysis (flavor and general acceptability) (*p* < 0.05). Compared to controls, the WHC% was increased in the breast meat of (G-M) birds (*p* < 0.05). Compared to controls, there were no effects of the probiotic supplementations on the pH, general sensory analysis (color, taste, chewiness, juiciness), or cooking loss of the breast meat (*p* > 0.05).

## 4. Discussion

Poultry welfare and meat quality characteristics are crucial concerns for the global poultry industry. After spending the growing phase in a controlled environment, the sudden exposure of birds to physiological stress, during their catching, handling, loading, motion, and acceleration negatively affects their well-being [13]. The interaction of these factors not only causes economic losses due to mortality and live weight shrinkage but can also deteriorate meat quality traits [13].

These alterations have a negative effect on neurons and their function, causing brain ultrastructural damage and dysfunction, including mental and emotional disorders expressed at both the immune and fear responsive levels [9]. The present results suggest that dietary probiotic supplementation improves the immune response and some meat quality traits in Muscovy ducklings. In the current study, the BW of the (G-A)-fed birds was remarkably improved as compared to controls but no differences were reported in the (G-M) and (G-A-M) groups. Similar results of BW change have been reported previously [7]. In our study, the average BW was 3783.60 g (control group), 3929.71 g (G-A), 3818.03 g (G-M), and 3791.10 g (G-A-M).

Similarly, improved performance has been reported in ducklings fed a diet with probiotics (*Saccharomyces cerevisiae*) [6]. However, whether the strain of bacteria has similar functions will be tested in future studies.

Probiotic administration has been used as a biotherapy for a variety of diseases, including anxiety- and depressive-like behaviors in rats and humans and cognitive impairment [14]. Studies have examined probiotics’ effect on neurotransmitter signaling and neuronal activation in the brain, as well as modulation of hormones and other chemicals in the body such as cytokines [14]. Several *Lactobacillus* and *Bifidobacterium* species are known to contribute significantly to innate immunity by boosting natural killer cell activity and macrophage phagocytosis, while also modulating adaptive immune responses through interactions with enterocytes, dendritic cells, and T helper and regulatory T cells [15].

In the present study, the tonic immobility test was not affected by the dietary probiotic supplementation. Failure to observe any treatment effects could be linked to multiple factors, such as the duck’s age [7], the probiotic concentration, and the length of feeding time when the experiment was conducted [7]. The current findings suggest that the probiotic supplementation under the current condition may not be suitable to affect the tonic immobility test; however, research is being conducted to identify mechanisms by which gut microflora can communicate and influence central nervous system (CNS) functioning [15].

Immune cells and commensal microbes in the intestine constantly communicate and react to each other in a stable environment in order to maintain healthy immune activities [16]. For example: in poultry, these commensal microorganisms include genera such as *Lactobacillus*, *Bifidobacterium*, *Enterococcus*, *Ruminococcus*, *Faecalibacterium*, and *Clostridium* clusters, which are commonly found in the gastrointestinal tract of birds [1]. These microorganisms contribute to microbiome stability through several mechanisms, including competitive exclusion of pathogens, production of short-chain fatty acids (SCFAs), modulation of intestinal pH, stimulation of mucus production, and activation of local immune cells, all of which support intestinal homeostasis [1,2,7]. Immune system–microbiota cross-talk relies on a complex network of pathways that sustain the balance between immune tolerance and immunogenicity [16]. Probiotic bacteria can interact with and stimulate intestinal immune cells and commensal microflora to modulate specific immune functions and immune homeostasis. Growing evidence shows that probiotic bacteria present important health-promoting and immunomodulatory properties [16].

Good bacteria (probiotics) reshape the intestinal microbiota by stimulating the synthesis of immunoglobulins [10], reducing the pH of the intestine [17], and preventing gut barrier permeability (Leaky gut) through improvement in the intestinal integrity and epithelial defense response [18].

In the present experiment, compared to controls, the probiotic-fed Muscovy ducks had a significantly higher count of *Lactobacilli* spp., which has a beneficial effect on stimulating the immune response. The ability for probiotic supplementations to have a protective effect on the gut microbiota may be attributed to the different types of good bacteria provided to the bird; the host’s species, age, strains, and health status; and the duration of the supplementation [2].

The specific interaction among the intestinal immune system and beneficial microbiota may enhance a signaling cascade in terms of pro- and anti-inflammatory cytokine production, which may improve immune function [19]. Evidence indicates that different groups of beneficial microbiota can enhance the inflammatory response by acting as immunoregulatory effectors [19]. Beneficial bacteria can therefore enhance an innate, non-specific, immune response in which innate immune cells locally regulate tissue injury [20]. Interestingly, compared to controls, the probiotic-supplemented birds showed a lower concentration of IL-10 in the spleen, but the reduction was observed only in the G-A group in the liver; at the same time, the concentration of the IL-6 was reduced in the G-A and G-A-M in the spleen and liver.

Modulation of the immune function can be linked with the presence of commensal microbiota in the gut, which ensures mechanical and structural integrity as well as the barrier function of intestinal mucosal surfaces, thus protecting the intestine, which in turn maintains a healthy immune system. Indeed, numerous beneficial bacteria species colonize in the intestine and play an important role in immune gut homeostasis [21].

Modification of diets has become a beneficial management tool for improvement in meat attributes in poultry [22,23]. The value of meat quality can be determined based on meat pH, water-holding capacity (WHC%), cooking loss (CL%), and tenderness “sensory attribute” [8]. Several studies have demonstrated that supplementing poultry diets with probiotics such as *Clostridium butyricum* [24] and *Saccharomyces cerevisiae* [25] can improve meat quality, particularly in terms of CL%, WHC%, overall sensory traits, and pH.

In the present study, breast muscle flavor, overall acceptability, and WHC% were significantly improved in the (G-M) group compared to the control. However, probiotic supplementation did not significantly affect the pH value of duck breast meat relative to the control group. This aligns with findings by [26], who also reported no significant change in the pH of Pegagan duck meat following dietary supplementation with lactic acid bacteria.

Meat quality is influenced by pH levels during the post-slaughter conversion of muscle to meat (rigor mortis), with a decrease in pH during this phase enhancing meat tenderness [27]. The average pH values measured 24 h after slaughter ranged from 5.98 to 6.07 across both control and treatment groups. These values are within the reported pH range for Muscovy duck breast meat (5.9–6.15) [28] and are comparable to those found by [29], who reported a pH range of 6.07–6.14 for the same type of meat.

Water-holding capacity (WHC) is a key meat quality trait closely associated with tenderness, which is considered the most important sensory attribute of meat [30]. The current study showed that (G-M) ducklings exhibited an increased WHC%, which contributes to better moisture retention and enhanced meat tenderness [31]. The improvement in WHC% was linked to elevated levels of omega-3 fatty acids, particularly docosahexaenoic acid (DHA) and eicosapentaenoic acid (EPA), which are known to support meat tenderness [24].

Additionally, previous research has shown that dietary supplementation with the probiotic *Bacillus subtilis* B2A enhanced WHC% in the breast meat of grower chicks [9]. On the other hand, the improvement in WHC% of duck breast meat in the G-A and G-A-M groups was not statistically significant compared to the control group. This finding is consistent with the results reported by [26], who observed no significant change in WHC% in Pegagan duck meat following dietary supplementation with lactic acid bacteria.

The relationship between pH and WHC% suggests that higher meat pH values are associated with a more compact muscle structure, resulting in greater water-holding capacity. In contrast, lower pH values lead to a more open muscle structure, reducing WHC% [32]. This trend is largely consistent with the findings of the present study.

Cooking loss (CL%) is closely associated with water-holding capacity (WHC); a lower CL% indicates reduced water and nutrient loss during cooking, which is generally linked to better meat quality [33]. Meat with low cooking shrinkage retains more moisture and nutritional value, contributing to overall quality. However, in the present study, no significant differences in CL% were observed between the treatment and control groups, showing a somewhat inconsistent relationship with the observed improvements in WHC%.

A similar inconsistency was reported in a study examining the technological and organoleptic qualities of Muscovy duck meat at different ages [28]. Although not statistically significant (*p* > 0.05), an increase in fat content in broiler chicken breast meat was observed following dietary supplementation with *Bacillus subtilis* [33]. This was related to variations in fat content, where an increase (*p* > 0.05) in fat content in broiler chicken breast meat was observed following dietary supplementation with *Bacillus subtilis* [32]. Additionally, a positive correlation has been reported between muscle lipid content and cooking loss in duck breast meat [34].

In comparison to the current study, lower percentages of WHC% (72.6–83.5%) and CL (36.1–40.7%) were reported by [29] for Muscovy duck breast meat raised under two different management systems.

In sensory assessment, the human sense organs function as detectors, transmitting information about food properties from external stimuli to the brain. They offer insights that no instrument can replicate, providing detailed evaluations of how a food item is perceived and the degree to which it is liked by consumers [35]. Among sensory attributes, texture, flavor, and overall appearance are considered the most critical factors influencing consumer preferences for meat [36]. In the present study, flavor and overall acceptability of the breast muscle were significantly enhanced in the (G-M) group compared to the control group. However, other sensory traits and treatment groups did not show significant improvements. This aligns with findings from [26], where no enhancement in meat tenderness was observed in Pegagan ducks fed a probiotic-supplemented (lactic acid bacteria) diet.

Furthermore, several studies have reported no significant effects on the sensory properties of breast meat in broiler-fed diets containing *Lactobacillus* spp., *Bifidobacterium* spp., *Lactococcus* spp., *Streptococcus thermophilus*, *Bacillus subtilis*, *Rhodopseudomonas* spp., and *Saccharomyces cerevisiae* yeast [37]. In contrast, other studies have demonstrated that dietary supplementation with probiotics such as *Lactobacillus acidophilus* and *Streptococcus cerevisiae* [38], or a combination of *Bacillus licheniformis* and *Bacillus subtilis* [39], can enhance broiler meat quality and sensory characteristics.

The improvement in sensory properties observed in probiotic-fed birds may be attributed to the ability of probiotics to inhibit the degradation of subcutaneous and intramuscular fat, which directly influences both flavor and overall acceptability [40]. It has been noted that myoglobin and hemoglobin levels, which are higher in darker meats such as duck, can accelerate lipid oxidation. Additionally, duck meat is particularly prone to oxidation due to its high content of unsaturated fatty acids, often resulting in the development of off-flavors and rancid odors during storage [41].

Moreover, poultry meat tenderness is influenced by several factors, including the quality of connective tissue (collagen), the integrity of the myofibrillar structure, and the interaction between muscle fibers and the extracellular matrix [28]. Improvement in flavor was linked to better muscle fiber structure in probiotic-fed Muscovy ducks, supporting the enhancement in sensory quality [42].

## 5. Conclusions

Taken together, the current findings indicate that dietary supplementation with a probiotic (*Bacillus subtilis* and *Bacillus licheniformis* strains) may be an alternative management strategy for efficiently improving Muscovy duck production performance and health, and further research is needed to improve meat quality traits.

## Figures and Tables

**Table 1:** Components of base diet ^1^.

Ingredient %	Starter (1–21 d)	Grower (22–60 d)
Physical Composition		
Yellow corn ground	46.00	33.00
Soybean meal 46%	31.00	20.00
Barley	10.00	20.00
Rice bran		15.00
Wheat middling	6.20	7.45
Soybean oil	2.00	
Limestone	1.25	1.25
Dicalcium phosphate	1.10	0.90
DL-Methionine	0.15	0.10
Sodium chloride	0.30	0.30
^2^ Vitamin and trace mineral premix	2.00	2.00
Calculated Analysis		
Crude protein %	20.02	17.10
Calcium %	0.93	0.85
Phosphorus%	1.02	0.86
Available phosphorus %	0.45	0.37
Methionine %	0.78	0.66
Methionine + Cystine %	0.65	0.67
Lysine %	0.40	0.34
Metabolizable energy MJ/Kg	12.20	11.56

^1^ The ration formulation was produced according to NRC (1994). A regular diet (G-C) without any supplementation of probiotics, G-A; supplemented with 0.4 g/kg of Amnil^®^, G-M; supplemented with 0.5 g/kg of M-Mobilize^®^, and G-A-M; supplemented with 0.4 g/kg of Amnil^®^ (1–30 day) and 0.5 g/kg of M-Mobilize^®^ (31–60 day), respectively. ^2^ Vitamin and trace mineral premix provided the following per kilogram of diet: Vitamin A: 12,000 IU, Vitamin D3: 5000 IU, Vitamin E: 130 mg, Vitamin K3: 3.6 mg, Vitamin B1: 3 mg, Vitamin B2: 8 mg, Vitamin B6: 4.95 mg, Vitamin B12: 0.17 mg, niacin: 60 mg, folic acid: 2.10 mg, biotin: 200 mg, calcium *D*-pantothenate: 18.3 mg, copper: 80 mg, iodine: 2 mg, selenium: 150 mg, iron: 80 mg, manganese: 100 mg, zinc: 80 mg, Se: 0.15 mg.

**Table 2:** Effect of dietary supplementation of multi-strain probiotic supplements on tonic immobility test of Muscovy ducks.

Treatment ^1^	(G-C)	(G-A)	(G-M)	(G-A-M)	SEM	*p*-Value
Tonic immobility test TI (sec)	224.16	209.83	178.00	172.83	58.39	0.9084

^1^ Dietary treatments containing a control (G-C) without any supplementation of probiotics, G-A; supplemented with 0.4 g/kg of Amnil^®^, G-M; supplemented with 0.5 g/kg of M-Mobilize^®^, and G-A-M; supplemented with 0.4 g/kg of Amnil^®^ (1–30 day) and 0.5 g/kg of M-Mobilize^®^ (31–60 day), respectively, *n* = 12 per treatment (pen was considered the experimental unit, and all two birds/pen were used to measure tonic immobility test and averaged for analysis).

**Table 3:** Effect of dietary supplementation of multi-strain probiotic supplements on immune response of Muscovy ducks.

Treatment ^1^	(G-C)	(G-A)	(G-M)	(G-A-M)	SEM	*p*-Value
Spleen						
Interleukin (IL)-6 S (pg/mL)	0.38 ^a^	0.24 ^b^	0.36 ^a^	0.21 ^b^	0.02	0.0001
Interleukin (IL)-10 S (pg/mL)	0.45 ^a^	0.14 ^b^	0.19 ^b^	0.15 ^b^	0.01	0.0001
Tumor Necrosis Factor (TNF)-α (pg/mL)	0.18	0.17	0.17	0.17	0.01	0.8255
Liver						
Interleukin (IL)-6 L (pg/mL)	0.48 ^a^	0.29 ^c^	0.41 ^ab^	0.38 ^b^	0.02	0.0001
Interleukin (IL)-10 L (pg/mL)	0.17 ^a^	0.13 ^b^	0.17 ^a^	0.17 ^a^	0.01	0.0074
Tumor Necrosis Factor (TNF)-α (pg/mL)	0.25	0.24	0.21	0.20	0.01	0.0589

^a,b,c^ Mean ± SEM with different superscripts in the same row differ significantly (*p* < 0.05). ^1^ Dietary treatments containing a control (G-C) without any supplementation of probiotics, G-A; supplemented with 0.4 g/kg of Amnil^®^, G-M; supplemented with 0.5 g/kg of M-Mobilize^®^, and G-A-M; supplemented with 0.4 g/kg of Amnil^®^ (1–30 day) and 0.5 g/kg of M-Mobilize^®^ (31–60 day), respectively, *n* = 12 per treatment (pen was considered the experimental unit, all two birds/pen were used to measure immune response and averaged for analysis).

**Table 4:** Effect of dietary supplementation of multi-strain probiotic supplements on cecal microbial ecology of Muscovy ducks.

Treatment ^1^	(G-C)	(G-A)	(G-M)	(G-A-M)	SEM	*p*-Value
*Lactobacillus* sp. (log 10 cfu/g)	6.47 ^b^	7.82 ^a^	7.62 ^a^	8.18 ^a^	0.28	0.0022
Coliforms (log 10 cfu/g)	4.11	5.21	4.34	3.97	0.36	0.1151

^a,b^ Mean ± SEM with different superscripts in the same row differ significantly (*p* < 0.05). ^1^ Dietary treatments containing a control (G-C) without any supplementation of probiotics, G-A; supplemented with 0.4 g/kg of Amnil^®^, G-M; supplemented with 0.5 g/kg of M-Mobilize^®^, and G-A-M; supplemented with 0.4 g/kg of Amnil^®^ (1–30 day) and 0.5 g/kg of M-Mobilize^®^ (31–60 day), respectively, *n* = 12 per treatment (pen was considered the experimental unit, and all two birds/pen were used to measure cecal microbial ecology and averaged for analysis).

**Table 5:** Effect of dietary supplementation of multi-strain probiotic supplements on meat quality of Muscovy ducks.

Treatment ^1^	(G-C)	(G-A)	(G-M)	(G-A-M)	SEM	*p*-Value
Breast						
pH	5.98	6.05	6.07	6.06	0.03	0.2035
Color	7.86	7.71	7.95	7.80	0.09	0.3222
Taste	8.33	8.02	8.33	8.21	0.09	0.0985
Texture	8.15	7.56	8.22	7.88	0.18	0.0645
Juiciness	8.30	7.35	8.25	7.79	0.18	0.0877
Flavor	7.63 ^b^	8.08 ^ab^	8.29 ^a^	7.98 ^ab^	0.13	0.0122
General acceptability	7.73 ^b^	8.14 ^ab^	8.21 ^a^	7.93 ^ab^	0.11	0.0288
Water-holding capacity (WHC) %	84.94 ^b^	85.87 ^ab^	86.28 ^a^	85.62 ^ab^	0.26	0.0109
Cooking Loss (CL) %	49.94	49.78	50.84	50.97	0.72	0.5605

^a,b^ Mean ± SEM with different superscripts in the same row differ significantly (*p* < 0.05). ^1^ Dietary treatments containing a control (G-C) without any supplementation of probiotics, G-A; supplemented with 0.4 g/kg of Amnil^®^, G-M; supplemented with 0.5 g/kg of M-Mobilize^®^, and G-A-M; supplemented with 0.4 g/kg of Amnil^®^ (1–30 day) and 0.5 g/kg of M-Mobilize^®^ (31–60 day), respectively, *n* = 12 per treatment (pen was considered the experimental unit, and all two birds/pen were used to measure meat quality parameters and averaged for analysis).

## Data Availability

The original contributions presented in this study are included in the article. Further inquiries can be directed to the corresponding author.

## Abbreviations

The following abbreviations are used in this manuscript:

GDP	Gross Domestic Product
IL-6	Interlukin-6
IL-10	Interlukin-10
TNF-α	Tumer Necrotic Factor Alpha
WHC%	Water-Holding Capacity %
CL	Cooking Loss
BW	Body Weight
CNS	Central Nervous System
DHA	Docosahexaenoic Acid
EPA	Eicosapentaenoic Acid
